# High titer heterologous rhamnolipid production

**DOI:** 10.1186/s13568-016-0298-5

**Published:** 2016-12-12

**Authors:** Janina Beuker, Theresa Barth, Anke Steier, Andreas Wittgens, Frank Rosenau, Marius Henkel, Rudolf Hausmann

**Affiliations:** 1 Department of Bioprocess Engineering (150k), Institute of Food Science and Biotechnology, University of Hohenheim, Fruwirthstr. 12, 70599 Stuttgart, Germany; 2 Ulm Center for Peptide Pharmaceuticals (U-PEP), Ulm University, Albert-Einstein-Allee 11, 89081 Ulm, Germany; 3 Evonik Industries, Evonik Technology and Infrastructure GmbH, Rodenbacher Chaussee 4, 63457 Hanau-Wolfgang, Germany

**Keywords:** Heterologous rhamnolipid biosynthesis, *Pseudomonas putida*, Biosurfactant, Fed batch cultivation

## Abstract

Heterologous mono-rhamnolipid production by *Pseudomonas putida* KT2440 pSynPro8oT_*rhlAB* using glucose as the single carbon source was characterized in fed-batch bioreactor cultivations. For the described experiments, a defined mineral salt medium was used, and a two phase glucose feeding profile was applied, which yielded a final rhamnolipid concentration of 14.9 g/L. Applying the feeding profile, glucose stayed almost constant until 28 h of cultivation and decreased afterwards to limiting levels. Until the end of cultivation 253.0 ± 0.1 g glucose was added to the bioreactor of which a total of 252.0 ± 0.6 g glucose was metabolized. By modeling the fed-batch bioreactor cultivations the time courses of generated biomass, rhamnolipid and consumed glucose were described. The model was furthermore used to derive key process parameters from the collected data. The obtained values for the specific product formation rates (q_RL_) reached 18 mg/(g h) and yield coefficients (Y_RL/S_) 10 mg/g respectively.

## Introduction

Low molecular weight amphiphilic compounds secreted by several microorganisms are often termed biosurfactants. These microbial surfactants exhibit diverse structures, are generally assumed to be easily biodegradable, to display a non-toxic character and often show bio-active properties (Hausmann and Syldatk 2014). Despite these advantages microbial surfactants are so far not used in large scale industrial production. Several specialized bioreactor concepts for the biosurfactant production have been reviewed by Beuker et al. (2014). The glycolipid rhamnolipid is one of the most studied microbial surfactants (Abdel-Mawgoud et al. 2011).

The heterologous and glucose-based rhamnolipid production brings several advantages and has been comparatively extensively studied since the 1990s. Firstly the pathogenicity and the complex biosynthesis regulation of the natural producer *Pseudomonas aeruginosa* can be avoided. Secondly glucose represents a convenient carbon source that avoids the difficulties in using vegetable oil used as a carbon source for *P. aeruginosa* (Müller and Hausmann 2011). However, reported heterologous rhamnolipid production with maximal product concentration of 7.3 g/L (Cha et al. 2008) is by far not convincing in comparison to that reported from *P. aeruginosa*. As stated before, heterologous rhamnolipid production in *Pseudomonas putida* KT2440 is not naturally regulated by *quorum sensing* leading to constitutive rhamnolipid biosynthesis. However, the actual handling of *P. putida* rhamnolipid producing strains remains challenging. This highly complex bacterial process exhibits multilayered interactions between glucose metabolization, biomass growth and product formation. These interactions impose difficulties to determine influences of changes in glucose feed on the cultivation. To encounter these difficulties model based process optimization with several iteration steps has been successfully employed in the past (Kovárová-Kovar et al. 2000).

Wittgens et al. (2011) examined heterologous rhamnolipid production using *P.* *putida* KT42C1 pVLT31_*rhlAB* in glucose enriched LB medium in a baffled shake flask experiment. In their study Wittgens et al. (2011) described biomass growth using a logistic equation and glucose metabolization as well as rhamnolipid formation using biomass dependent ordinary differential equations (ODEs). In their approach rhamnolipid productivity as well as glucose metabolization exhibited constant values and were not growth rate dependent. *P. putida* KT42C1 pVLT31_*rhlAB* was reported to produce up to 1.5 g/L of rhamnolipid.

## Methods

Methods were adapted from foam fractionation processes as described in Beuker et al. (2016).

### Chemicals

All chemicals used in the current study were purchased from Carl Roth GmbH (Karlsruhe, Germany) if not stated otherwise.

### Microorganism and plasmid

*P. putida* KT2440 with plasmid pSynPro8oT_*rhlAB* producing mono-rhamnolipids was used as described in Beuker et al. (2016).

### Culture conditions

#### Media

Tetracycline was added to all media to an end concentration of 20 mg/L. For the first culture LB medium (5 g/L yeast extract (BD, Heidelberg, Germany), 10 g/L tryptone (BD), 5 g/L NaCl; pH 7.0) was utilized. For seed culture SupM medium (4.4 g/L Na_2_HPO_4_∙2 H_2_O, 1.5 g/L KH_2_PO_4_, 1 g/L NH_4_Cl, 0.2 g/L MgSO_4_∙7 H_2_O, 0.02 g/L CaCl_2_∙2 H_2_O, 0.006 g/L FeCl_3_, 30 g/L glucose, 10 g/L yeast extract, 1 mL/L trace element solution 2, pH 6.8; trace element solution 2: 0.3 g/L H_3_BO_3_, 0.2 g/L CoCl_2_∙6 H_2_O, 0.1 g/L ZnSO_4_∙7 H_2_O, 0.03 g/L MnCl_2_∙4 H_2_O, 0.01 g/L CuCl_2_∙2 H_2_O, 0.03 g/L Na_2_MoO_4_∙2 H_2_O, 0.02 g/L NiCl_2_∙6 H_2_O) was applied. In the bioreactor cultivation ModR medium (22 g/L KH_2_PO_4_, 2.6 g/L (NH_4_)_2_HPO_4_, 1.4 g/L MgSO_4_∙7 H_2_O, 0.87 g/L citric acid, 0.01 g/L FeSO_4_∙7 H_2_O, 5 g/L glucose, 10 mL/L trace element solution 2, pH 6.8) was used. 15 g/L antifoaming agent Tego KS 53 (Evonik Industries, Essen, Germany) was added to the bioreactor cultivation. Feed medium contained 400 g/L glucose, 22 g/L KH_2_PO_4_, 3.43 g/L KH_2_PO_4_, 0.01 g/L FeSO_4_∙7 H_2_O and 0.1∙10^−3^ g/L CuCl_2_∙2 H_2_O.

#### Preparation of seed culture

All shake flasks were inoculated in a shake incubator chamber (Multitron II, HT Infors, Bottmingen, Switzerland) at 30 °C and 120 rpm. First 25 mL LB in a 100 mL baffled shake flask were inoculated with 50 µL from a glycerol stock solution of *P. putida* KT2440 pSynPro8oT_*rhlAB* and incubated for 24 h. Seed cultures contained 100 mL SupM medium in a 1 L baffled shake flask inoculated with 1 mL from the 24 h LB culture and incubated for 12 h.

#### Bioreactor cultivations

All bioreactor cultivations were carried out as duplicates. The bioreactor (Minifors, HT Infors, Bottmingen, Switzerland) was equipped with an integrated pH, temperature and aeration control system. During bioreactor cultivation aeration was set to 0.133 vvm and pO_2_ was controlled at 13% via stirring rate starting with a minimum of 300 rpm. Temperature was held constant at 30 °C and pH was controlled to 6.8 via 1 M H_2_SO_4_ or 19% NH_4_OH. Generated foam was detected by an antifoam probe and antifoaming agent Tego KS 53 (Evonik Industries) was added if needed. Bioreactors were inoculated with 12 h SupM seed culture to a final OD of 0.5. Initially 6 g glucose was provided in the culture vessel. After 5 h of batch fermentation a dual phase feeding profile was started as displayed in Fig. 2b.

### Analytical methods

#### Sampling and processing

Taken bioreactor samples were mixed with equal volumes of hexane and centrifuged (4700 rpm, 15 min, 4 °C) to remove antifoaming agent. The hexane phase was discarded and the cell free aqueous supernatant was used for rhamnolipid, glucose and ammonium detection. The cell pellet was washed with 1:1 (v/v) 9 g/L NaCl solution, centrifuged (4700 rpm, 15 min, 4 °C), dried at 100 °C and used for gravimetrical cell dry weight determination.

Rhamnolipid detection was performed as described by Schenk et al. (1995) with minor adjustments. Part of the liquid phase was acidified with 1:100 (v/v) phosphoric acid and rhamnolipids were extracted twice with 1.25:1 (v/v) ethyl acetate. For rhamnolipid measurement appropriate amount of this ethyl acetate extract was evaporated. Rhamnolipids were resolved in acetonitrile and derivatized for 90 min at 1400 rpm and 60 °C using a 1:1 mixture of 40 mM bromphenacylbromid and 20 mM tri-ethyl-ammonium/-amin. Detection of rhamnolipids was performed using a HPLC device (Agilent 1100 Series, Agilent, Waldbronn, Germany) equipped with a 15 cm reversed phase column (Supelcosil^®^ LC-18, Supelco, Deisenhofen, Germany) at 30 °C. The mobile phase was composed of 100% methanol and ultrapure water. For rhamnolipid detection a gradient was applied. During the first 17 min methanol concentration was increased starting at 80 to 100%. This methanol concentration was held for 8 min and decreased to 80% during the next 5 min. Rhamnolipids were detected at a wave length of 254 nm at 30 °C. For calibration standard solutions of rhamnolipid in acetonitrile were used.

The concentration of glucose and ammonium were detected from the aqueous phase of samples using glucose (R-Biopharm AG, Darmstadt, Germany) and ammonium (Merck KGaA, Darmstadt, Germany) assay kits, respectively, according to the manufacturers’ instructions.

### Software for modeling and parameter optimization

Modeling was performed in a mathematical/numerical computing environment (Matlab, The MathWorks, Natick, MA, USA). To solve the ordinary differential equations the numeric solver “ode45” embedded in Matlab environment was used based on an explicit Runge–Kutta formula. Parameter optimization was performed using the Matlab embedded function “lsqnonlin” applying a trust-region-reflective algorithm, which is based on the interior reflective Newton method. The parameter optimization was executed by minimizing the sum of squares of the errors between simulation data and measured data considering biomass, rhamnolipid and glucose.

### Equations for modeling

**Fig. 1:**
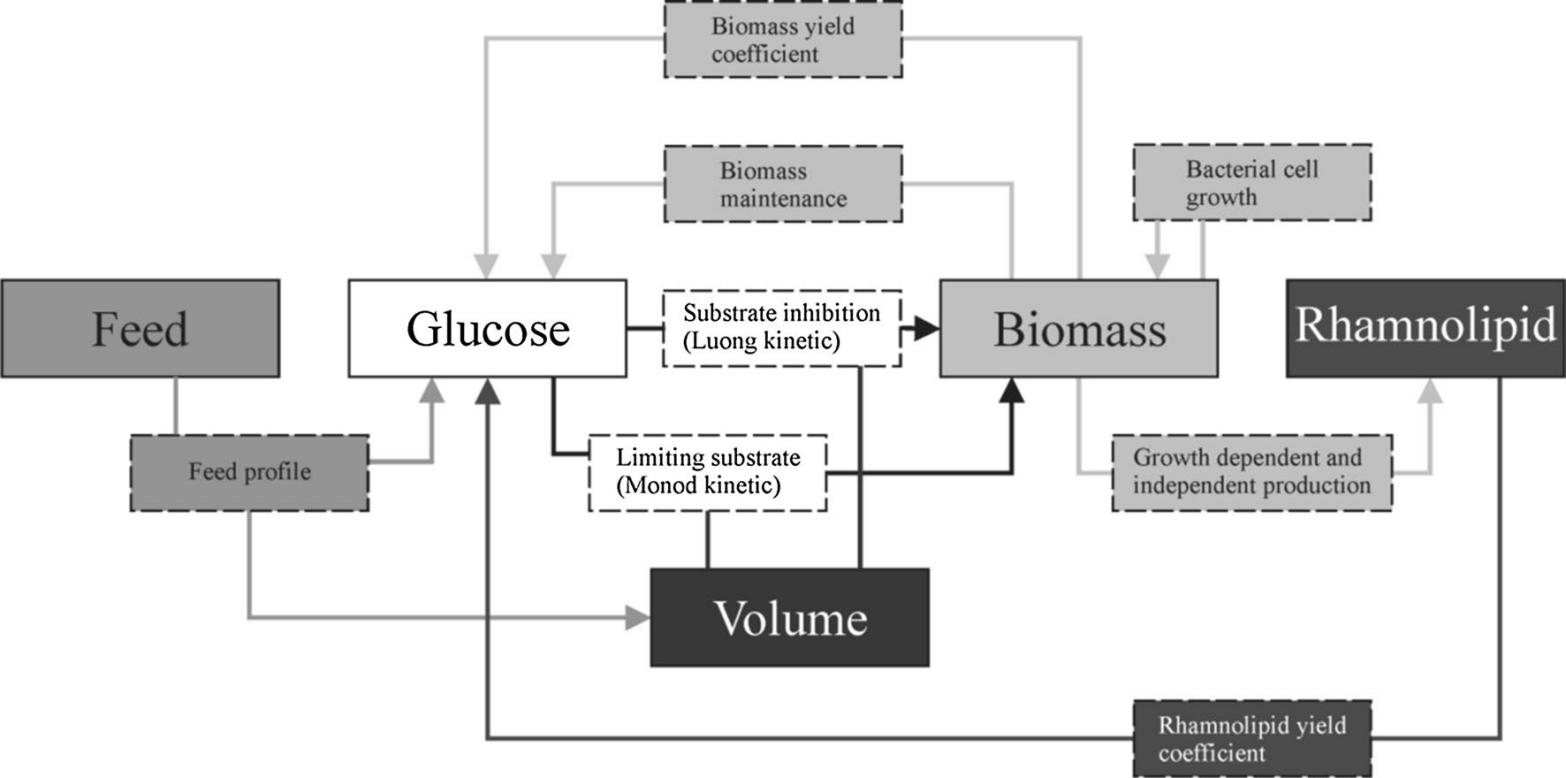
Dependencies between volume, feed, glucose, biomass and rhamnolipid; dependent parameter are depicted in *framed boxes*, *dashed boxes* depict type of dependencies, *arrows* show the source(s) and sink of dependencies

The general model setup is depicted in Fig. 1 outlining the interactions between glucose consumption, biomass growth, rhamnolipid production, feed and volume of the cultivation broth. All parameters used for modeling are summarized in Table 1.

**Table 1:** Parameters for modeling

Parameter	Meaning	Unit
α*	Growth dependent production term	–
β*	Growth independent production term	h^−1^
µ	Growth rate	h^−1^
µ_max_*	Maximal growth rate	h^−1^
ρ	Density	g L^−1^
τ*	Lag time	h
BR	Bioreactor	–
c_Index_	Concentration of index	g L^−1^
Glu	Glucose	g
K_i_*	Inhibiting concentration of glucose	g L^−1^
K_S_*	Monod constant	g L^−1^
${\dot{\text{m}}}$	Mass flux	g h^−1^
m_S/X_*	Maintenance coefficient	g g^−1^ L^−1^
n*	Shape factor for Luong kinetic	–
RL	Rhamnolipid	g
t	Time	h
V_Index_	Volume of index	L
X	Biomass	g
Y_P/S_*	Rhamnolipid yield coefficient	g g^−1^
Y_X/S_*	Biomass yield coefficient	g g^−1^

* Parameter determined via optimization

#### Biomass

Biomass mass growth was described via an autocatalytic process and a jump function to model lag times (Eq. 1). The jump function depended on the lag time (τ) and the autocatalytic process on existing biomass (X) and specific growth rate (µ).

1 $$\frac{\text{dX}}{\text{dt}} = \frac{1}{{1 + {\text{e}}^{{100 *\left( { - {\text{t}} + {\tau} } \right)}} }} * {\mu} * {\text{X}}$$

The specific growth rate was represented by a combination of Monod and Luong kinetic (Eq. 2). Unhindered growth was described by Monod kinetic depending on the concentration of the growth limiting substrate (c_Glu_), a Monod constant (K_S_) and a maximal growth rate (µ_max_) (Monod 1949). Inhibiting effects of high glucose concentration were integrated using Luong kinetic including the concentration of the growth limiting substrate, an inhibitory constant (K_i_) and a shape factor (n) (Luong 1987).

2 $${\mu} = {\mu}_{ \hbox{max} } *\left( {\frac{{{\text{c}}_{\text{Glu}} }}{{{\text{c}}_{\text{Glu}} + {\text{K}}_{\text{S}} }}} \right)*\left( {1 - \left( {\frac{{{\text{c}}_{\text{Glu}} }}{{{\text{K}}_{\text{i}} }}} \right)^{\text{n}} } \right)$$

Therefore, biomass growth was modeled using Eq. 3.

3 $$\frac{\text{dX}}{\text{dt}} = \frac{1}{{1 + {\text{e}}^{{100 *\left( { - {\text{t}} + {\tau} } \right)}} }}* {\mu}_{ \text{max} } *\left( {\frac{{{\text{Glu/V}}_{{{\text{BR}}}} }}{{{\text{Glu/V}}_{{{\text{BR}}}} + {\text{K}}_{\text{s}} }}} \right) *\left( {1 - \left( {\frac{{{\text{Glu/V}}_{{{\text{BR}}}} }}{{{\text{K}}_{{\text{i}}} }}} \right)^{{\text{n}}} } \right) *X$$

#### Glucose

The time course of glucose was modeled taking various glucose sinks as well as sources into account (Eq. 4). Glucose was assumed to be utilized by biomass and rhamnolipid generation as well as by maintenance of the biomass. Conversion efficiencies of glucose to biomass or rhamnolipid were determined by biomass and rhamnolipid yield coefficients (Y_X/S_ and Y_P/S_, respectively). Glucose amount used for maintenance was described by a maintenance coefficient (m_S/X_). Applied feed (${\dot{\text{m}}}_{\text{Feed}}$) with its glucose feed concentration (c_Glu in Feed_) was determined as glucose input.

4 $$\frac{\text{dGlu}}{\text{dt}} = {\dot{\text{m}}}_{\text{Feed}} *{\text{c}}_{\text{Glu in Feed}} - \left( {\frac{\text{dX}}{\text{dt}} *\frac{1}{{{\text{Y}}_{{{\text{X}}/{\text{S}}}} }} + \frac{\text{dRL}}{\text{dt}}*\frac{1}{{{\text{Y}}_{{{\text{P}}/{\text{S}}}} }} + {\text{m}}_{{{\text{S}}/{\text{X}}}} *{\text{X}}} \right)$$

#### Rhamnolipid

Rhamnolipid kinetic was described by a growth dependent (α) and growth independent (β) part of the specific biomass productivity (Eq. 5).

5 $$\frac{\text{dRL}}{\text{dt}} = \left( {{\alpha} * {\mu} + {\beta} } \right)*{\text{X}}$$

#### Bioreactor volume

For changes in the overall bioreactor volume just changes due to the feed medium were considered (Eq. 6). Volume addition by acid, base and antifoaming agent were assumed to level out by volume reduction due to sampling.

6 $$\frac{{{\text{dV}}_{\text{BR}} }}{\text{dt}} = \frac{{{\dot{\text{m}}}_{\text{Feed}} }}{{\rho} }$$

To describe the time course of the bioreactor cultivation the 10 parameter shown in Table 1 were optimized using the developed software. Optimization was implemented using measuring data of the double determination cultivation applying a dual phase feeding profile and the set of ODEs (Eq. 3, 4, 5, 6).

## Results

### Time courses of overall biomass, rhamnolipid and glucose during bioreactor cultivation

**Fig. 2:**
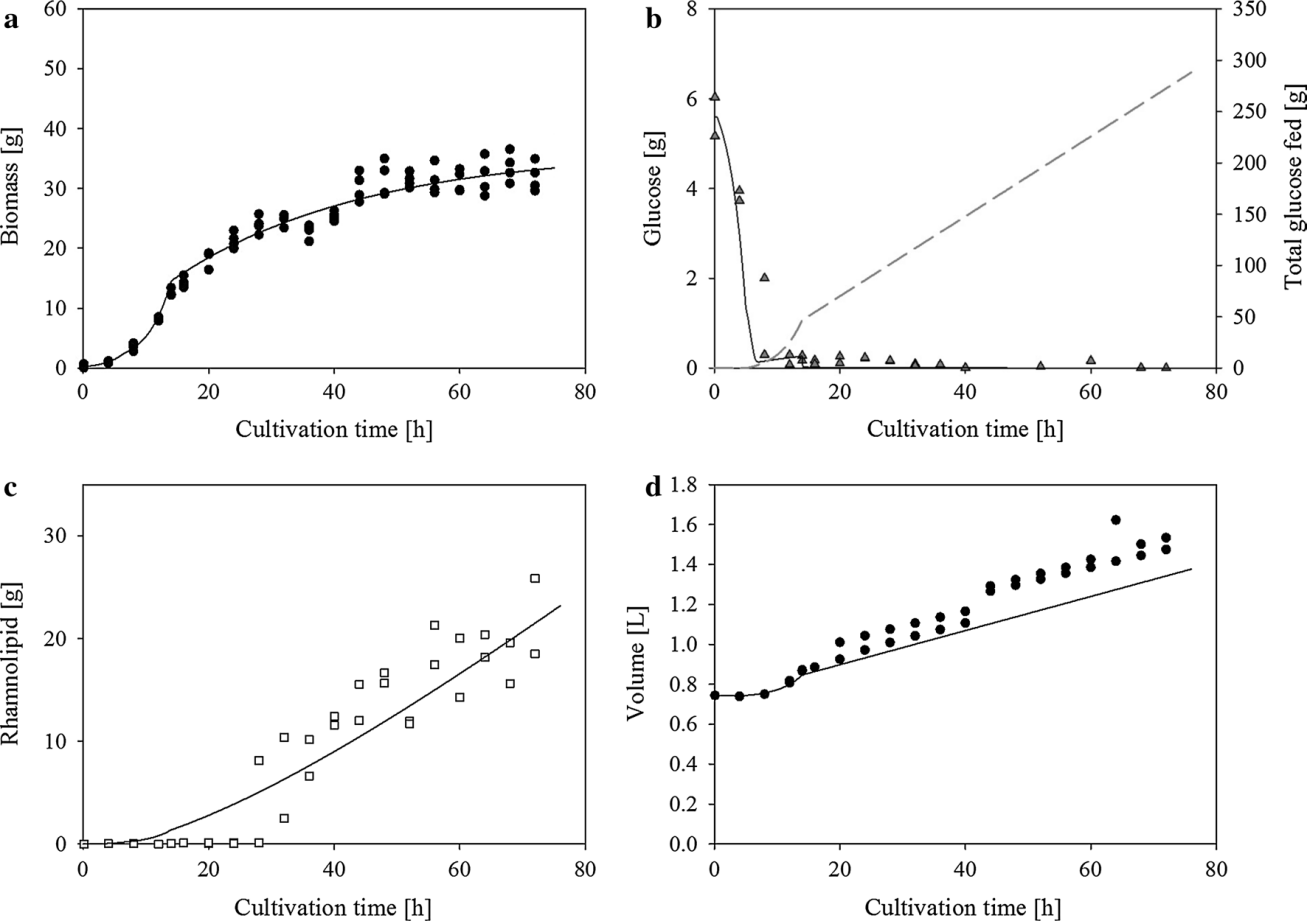
Time courses of *P. putida* KT2440 pSynPro8oT_*rhlAB* bioreactor cultivation measurement data compared to modeled data. **a** experimentally determined (*black circles*) and modeled total biomass time courses (*black line*), **b** experimentally determined (*grey triangles*), modeled glucose time courses (*black line*) and total fed glucose amount (*grey dashed line*), **c** measured rhamnolipid (*empty squares*) and modeled rhamnolipid time course (*black line*), **d** calculated bioreactor volume at each sampling point (*black circles*) and modeled bioreactor volume time course (*black line*) applying parameters depicted in Table 2

Time courses of generated biomass, rhamnolipid and consumed glucose are depicted in Fig. 2. In bioreactor cultivations applying the dual phase feeding profile glucose amount decreased in the beginning, stayed almost constant at 0.2 g from 12 h till 28 h and decreased afterwards to limiting levels. Biomass grew constantly with a leveling trend in the end reaching 35.3 ± 3.2 g in the end of cultivation. Rhamnolipid generation started after 24 h and increased steadily to a maximal value of 22.2 ± 4.3 g after 72 h, which equals a concentration of 14.7 ± 2.6 g/L. Until the end of cultivation 253.0 ± 0.1 g glucose was added to the bioreactor and in total 252.0 ± 0.6 g glucose was metabolized.

### Parameter optimization

To describe the time course of bioreactor cultivations a parameter optimization was conducted using the set of ODEs (Eq. 3, 4, 5, 6) and the experimental data. The results are depicted in Fig. 2 and Table 2.

**Table 2:** Optimized model parameters

Parameter	Optimized value	Unit
α	0.0477	–
β	0.0125	h^−1^
µ_max_	0.4386	h^−1^
τ	0.4686	h
K_i_	107.3604	g L^−1^
K_S_	0.2034	g L^−1^
m_S/X_	0.0599	g g^−1^ L^−1^
n	0.6167	–
Y_P/S_	0.2637	g g^−1^
Y_X/S_	0.3253	g g^−1^

Biomass and glucose time course were satisfactorily described using the model with the optimized parameter with end values of 33.5 and 0.0 g, respectively. However, rhamnolipid production was just described properly in the end of cultivation with a final rhamnolipid mass of 23.2 g which equals a concentration of 14.9 ± 0.5 g/L. The overall rhamnolipid production rate reached 0.3 g/h and the final production rate 0.4 g/h. The volume of the bioreactor was underestimated after 14 h, because modeled volume just considered changes caused by feeding medium.

**Fig. 3:**
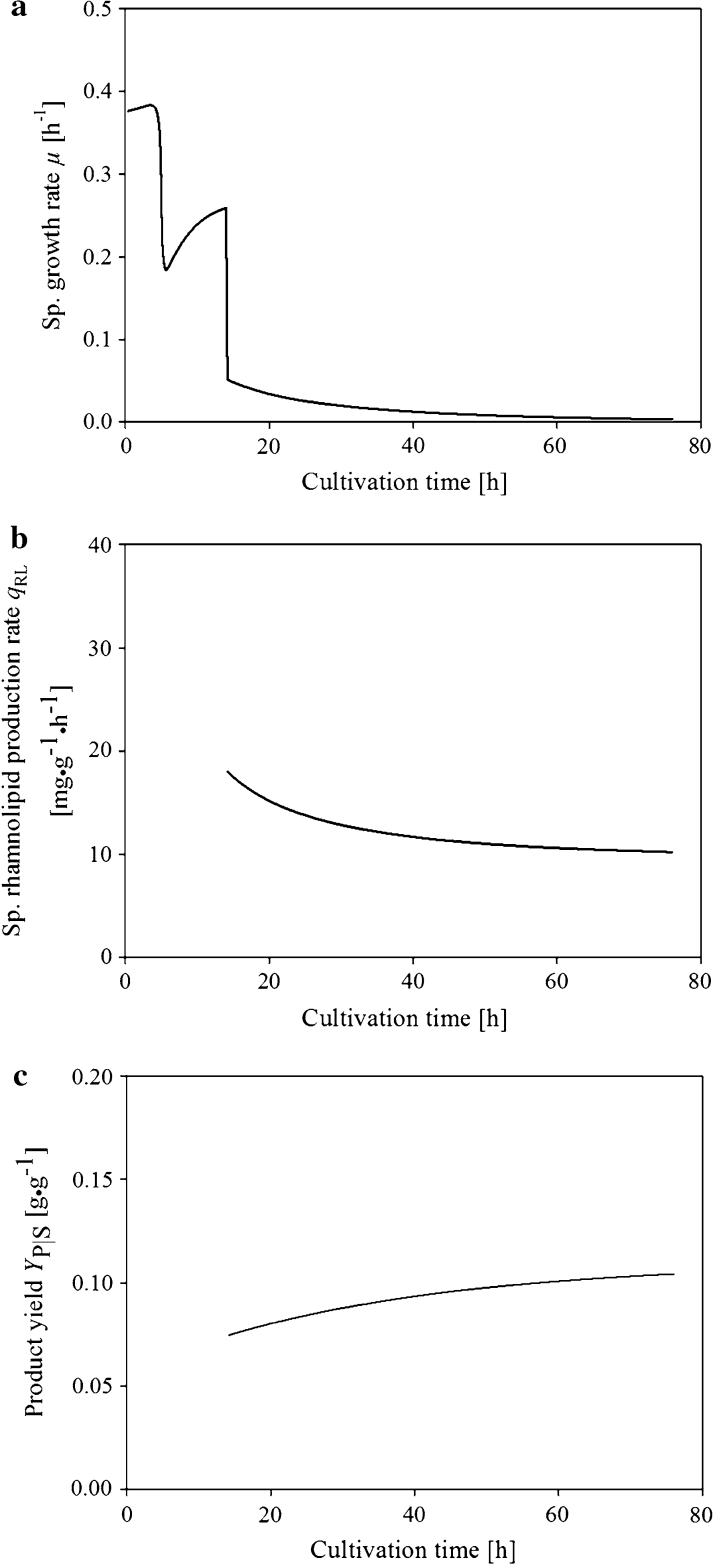
Time courses of modeled-derived process parameters for *P. putida* KT2440 pSynPro8oT_*rhlAB* bioreactor cultivation **a** time course of growth rate, **b** time course of specific rhamnolipid production rate, **c** time course of product yield

In Fig. 3 the model-derived time courses of the growth rate (µ), the specific rhamnolipid production rate (q_RL_) and the yield coefficient (Y_P/S_) are shown. The model-derived values for the growth rate show three distinct phases, reflecting the batch phase before 14 h and the fed-batch phase thereafter. The highest value for the growth rates in the batch phase were between 0.38 and 0.19 1/h while in the fed-batch phase values below 0.052 1/h were computed. Because rhamnolipid amounts in the fermentation broth accumulate only in the fed-batch phase after 14 h the values of the specific rhamnolipid production rate and the yield coefficient are only meaningful after that time. The highest value for the specific productivity (18 mg/(g h)) was registered immediately after switching to the feeding of glucose. Thereafter it steadily reduced its value until reaching 10 mg/(g h) at the end of the process. The product yield increased from 0.075 to 0.104 g/g during the fed-batch phase. This is an expected finding as the growth rates in the fed-batch phase decreased steadily from 0.052 to 0.002 1/h. It may be assumed that a reduced growth rate leads to a reduced glucose demand derived for cell growth. This may led to a better yield of product formation.

## Discussion

In this study a bioreactor fed-batch cultivation for heterologous rhamnolipid production in a defined medium and a process model was established. As a result up to 14.9 g/L rhamnolipid were obtained. Utilizing a dual phase feeding profile in bioreactor cultivations resulted in an overall rhamnolipid productivity of 0.3 g/h and rhamnolipid titer of 14.9 g/L. So far, in literature the highest rhamnolipid titer reported using heterologeous production hosts was 7.3 g/L (Cha et al. 2008). Therefore, the effectiveness of this bioreactor cultivation applying the feeding profile was demonstrated as well as the metabolic capacity of the construct *P. putida* KT2440 pSynPro8oT_*rhlAB*.

Differences in rhamnolipid amounts in experimental and modeled data were mainly caused by a lag time in rhamnolipid production in the beginning of cultivation. This lag time did not occur in the model. This lag time in rhamnolipid production could be caused by several reasons. One explanation could be the availability of one or both rhamnolipid precursors, which may be impaired or shifted at high growth rates thus resulting in a reduced specific production rate. It is noticeable that rhamnolipid production started when glucose started to be limiting corresponding to the onset of the glucose feeding. The recorded value of 18 mg/(g h) is comparable to the maximum value for the *P. putida* KT42C1 pVLT31_*rhlAB* reported by Wittgens et al. (2011) and about 38% for the very similar construct *P. putida* KT2440 pSynPro8_*rhlAB* grown on a complex rich medium containing additional glucose (Tiso et al. 2016). However, the comparison to experimental data reported on complex rich medium must be considered with caution.

The established model could be used to describe time courses of biomass and glucose during the overall bioreactor cultivation and of rhamnolipid in the end of bioreactor cultivation. Additionally the time courses of the growth rate, the specific productivity and the yield coefficient could be derived. To determine which factors influences biomass as well as rhamnolipid production further experiments should be conducted concerning medium components, e.g. the influence of iron and copper. Additionally, further investigations should be made to determine if other essential components should be added to the cultivation due to depletion by high biomass growth (e.g. sulphur, magnesium, calcium, sodium or trace elements).

For future work, strain engineering should focus on enhancing the effective yield that reached a value of 10% in relation to the theoretical maximum value of 49% (Henkel et al. 2012). Therefore, the putative generation of polysaccharides as byproducts should be mainly addressed. Another approach will be the combination of the strategy presented here using fed-batch processing, with in situ *product removal* by foam fractionation (Beuker et al. 2016) and thus avoiding the necessity of chemical antifoaming agents. This could lead to a further enhanced semi-continuous process.
